# Supplementary material for: Correlation of Pseudomonas aeruginosa Phage Resistance with the Numbers and Types of Antiphage Systems
Source: Int J Mol Sci. 2024 Jan 24;25(3):1424. doi: 10.3390/ijms25031424 (PMC10855318; doi:10.3390/ijms25031424)
Supplement: Supplementary file 1 [file ijms-25-01424-s001.zip › Supplementary Figure S1 - Prophage Analysis.pdf]

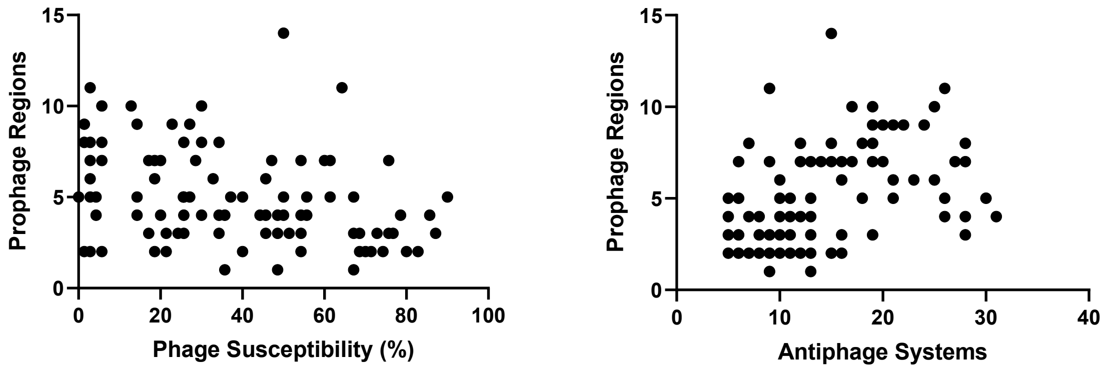

(a) (b)

Figure S1. Correlation of prophage regions with phage susceptibility and numbers of antiphage systems. (a). Correlation of prophage regions identified by PHASTER and strain susceptibility to phages. (b). Correlation of prophage regions with antiphage systems identified on the genome.
