# Supplementary material for: Correlation of Pseudomonas aeruginosa Phage Resistance with the Numbers and Types of Antiphage Systems
Source: Int J Mol Sci. 2024 Jan 24;25(3):1424. doi: 10.3390/ijms25031424 (PMC10855318; doi:10.3390/ijms25031424)
Supplement: Supplementary file 1 [file ijms-25-01424-s001.zip › Supplementary Figure S2 - Multivariate Analysis.pdf]

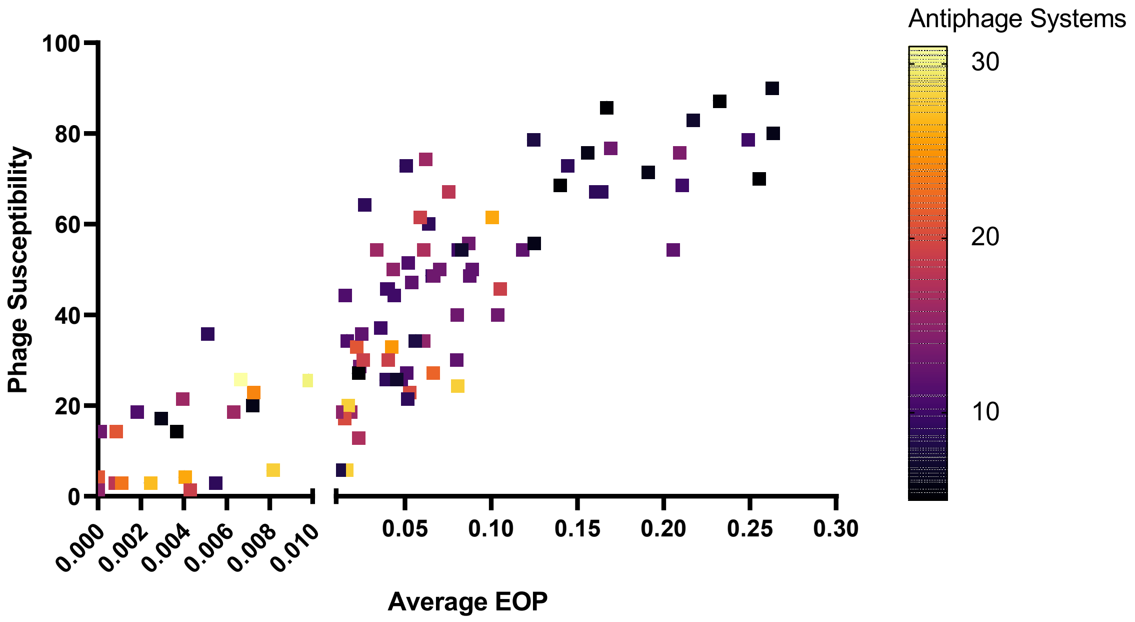

Figure S2. Multivariate analysis of 100 *P. aeruginosa* strains examining relationship between number of predicted antiphage systems with average efficiency of plating and overall phage susceptibility.
