# Supplementary material for: Correlation of Pseudomonas aeruginosa Phage Resistance with the Numbers and Types of Antiphage Systems
Source: Int J Mol Sci. 2024 Jan 24;25(3):1424. doi: 10.3390/ijms25031424 (PMC10855318; doi:10.3390/ijms25031424)
Supplement: Supplementary file 1 [file ijms-25-01424-s001.zip › Supplementary Figure S3 - Box and Whiskers Plot.pdf]

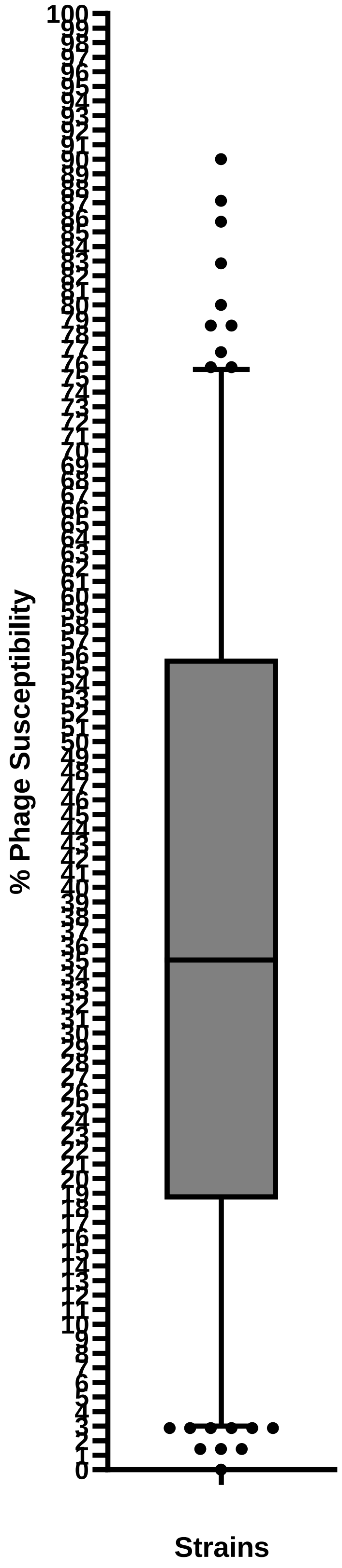
% Phage Susceptibility
Strains

Figure S3. Box and whiskers plot of 100 strains of *P. aeruginosa* showing median phage susceptibility and the 10 most extreme strains. 10 strains lysed by 75.5% or greater of the phages constitute the most phage susceptible, 10 strains lysed by fewer than 3% of the phages constitute the most phage resistant. 40 strains lysing more than 3% but less than 35% constitute intermediate resistant strains. 40 strains lysed by greater than 35% but less than 75.5% of phages constitute intermediate susceptible strains.
